# Movement Paradigm for Hazara Torrent Frog *Allopaahazarensis* and Murree Hills Frog Nanoranavicina (Anura: Dicroglossidae)

**DOI:** 10.3897/BDJ.10.e84365

**Published:** 2022-05-16

**Authors:** Ayesha Akram, Muhammad Rais, Muhammad Saeed, Waseem Ahmed, Sumbul Gill, Jibran Haider

**Affiliations:** 1 Herpetology Lab, Department of Zoology, Wildlife and Fisheries, Pir Mehr Ali Shah-Arid Agriculture University Rawalpindi, Rawalpindi 46000, Pakistan, Rawalpindi, Pakistan Herpetology Lab, Department of Zoology, Wildlife and Fisheries, Pir Mehr Ali Shah-Arid Agriculture University Rawalpindi, Rawalpindi 46000, Pakistan Rawalpindi Pakistan; 2 PMAS Arid Agriculture University Rawalpindi, Rawalpindi, Pakistan PMAS Arid Agriculture University Rawalpindi Rawalpindi Pakistan; 3 Gilgit-Baltistan Forest, Wildlife and Environment Department, Gilgit 15100, Pakistan, Gilgit, Pakistan Gilgit-Baltistan Forest, Wildlife and Environment Department, Gilgit 15100, Pakistan Gilgit Pakistan

**Keywords:** conservation, dispersal, endemic, frogs, habitat, radio telemetry

## Abstract

Endemic anurans are particularly vulnerable to environmental changes, and are susceptible to population declines because of their restricted distribution ranges. The Murree Hills Frog *Nanoranavicina* and Hazara Torrent Frog *Allopaahazarensis* are associated with the torrential streams and nearby clear water pools in subtropical chir pine forest and other forest types, at elevations higher than 1000 m in Pakistan. In this study, we have provided data on the extent of movement of these frog species for the first time. We installed radio transmitters on a total of 13 Murree Hills Frogs and 13 Hazara Torrent Frogs during eight consecutive days in September 2017 and 2018. Our results showed that these frogs did not move long distances along the stream or away from the stream into the forest. All the radio-tracked frogs showed movement of < 3 m. We found a significant differences only in the distance moved by Murree Hills Frogs between the two years studied. Based on our findings, we propose a movement paradigm that focuses on conservation implications for these endemic frogs.

## Introduction

Conservation of amphibians is becoming more vital due to the increasing global extinction rate in this group (Rais et al. 2021). Amphibians respond to geophysical characteristics at broad spatial scales, but few studies have examined their response to changes in landscape structure and climate change (Dupuis et al. 2000, Adams and Bury 2002). The ultimate structure of amphibian populations depends on the success of dispersal, spatial distribution of water bodies and connectivity of breeding sites (Semlitsch and Bodie 1998, Marsh and Trenham 2001, Moilanen and Nieminen 2002). Structural and functional landscape connectivity is essential for dispersal of species across the landscape (Taylor et al. 2006).

Studies on habitat use provide useful information for species conservation (Manly et al. 2002, Brunjes et al. 2006, Aarts et al. 2008). Endemic amphibians are particularly vulnerable to environmental changes and are susceptible to population declines (Lecis and Norris 2004, Moore et al. 2004) because of their restricted distribution range. The Murree Hills Frog and Hazara Torrent Frog are endemic to South Asia and Pakistan, respectively (Khan 2006, Rais et al. 2021). These frogs are associated with the torrential streams and nearby clear water pools in subtropical chir pine forest and Himalayan moist temperate forest at elevations higher than 1000 m (Ahmed et al. 2020), and their breeding season is from July-August (Saeed et al. 2021). The two species are categorized as Least Concern by the Red List of Threatened Species by the International Union for Conservation of Nature (Ahmed et al. 2020). Habitat degradation, urbanization and climate change are the known threats to these species (Ohler and Dutta 2004, Khan et al. 2008). Currently, there are no published data on the movement of these species. Given this, the present study used radio telemetry to assess for the first time the movements of *Allopaahazarensis* and *Nanoranavicina* and proposed a movement paradigm that focuses on conservation implications for these endemic frogs.

## Materials and methods

**Figure 1.**
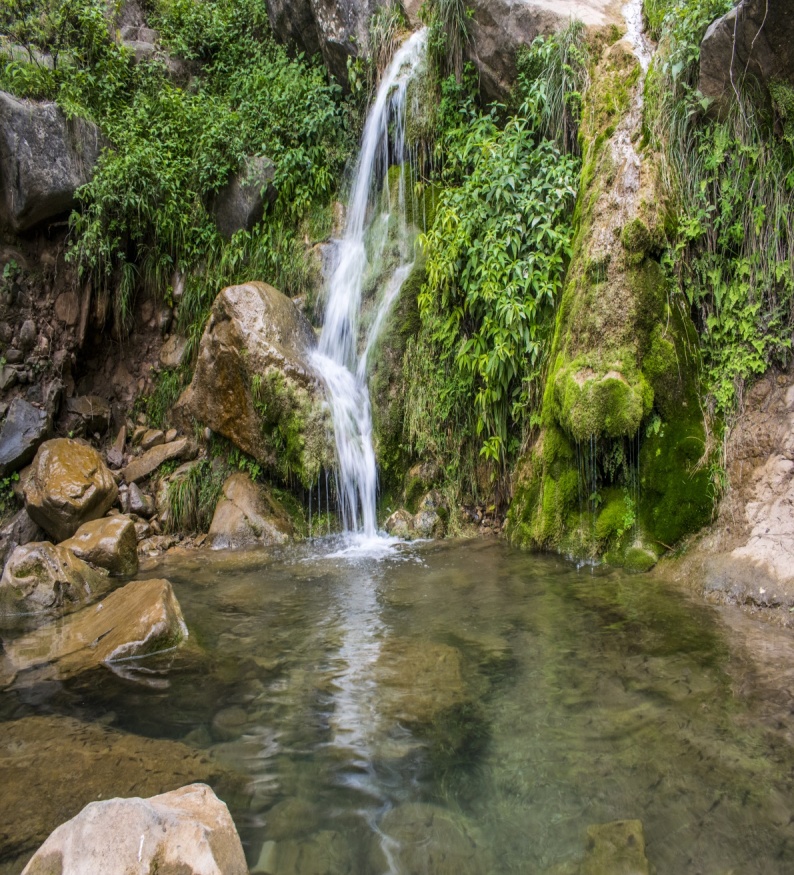
Freshwater stream at the Village Parhanna, Tehsil Murree, District Rawalpindi, Punjab Province, Pakistan. Photographed by Muhammad Saeed.

***Study area and species.***— We conducted the present study on Murree Hills Frog (*Nanoranavicina*) and Hazara Torrent Frog (*Allopaahazarensis*) at a natural freshwater stream (33.8432°N, 73.4694°E; 1693 m elevation), located in Village Parhanna, Tehsil Murree, District Rawalpindi, Punjab Province, Pakistan. This stream cascades over rapids and has a few associated ponds (Fig. 1). The stream is part of the Murree-Kotli Sattiyan and Kahuta National Park, Punjab, Pakistan. The topography of the National Park at higher altitude is mainly composed of rugged terrain with narrow valleys. The hilly area contains valleys created by the fast flowing running water of streams and rivers (Atta-ur-Rahman et al. 2010). Most of the vegetation in the area consists of sub-tropical chir pine forest and Himalayan moist temperate (Khan 2006).

*Installing radio transmitters.*—We captured the frogs using dip nets. We used Holohil BD-2A transmitters (0.49 g) and followed the attachment method by Muths (2003). We arranged the transmitter so that the battery was orientated towards the rear of the individual to allow it to move easily in the water. We made adjustments to the assembly system to make it easier for frogs to carry the transmitter. We constructed the radio transmitter belts by using a very thin, soft elastic thread and light-weight, brightly-coloured plastic beads. For each assembly, we placed a transmitter in the center of the elastic thread, with an equal number of beads on both sides of the transmitter and tied a knot in the elastic thread. We ensured that the belt was not so tight that it constricted the frog’s body and not so loose that it could easily slip off. To attach the transmitter, we stretched the legs of the frog and adjusted the belt as needed to fit around the frog’s waist (Muths 2003) (Fig. 2). We used a vernier caliper (Insize Precision Measurement Vernier Caliper SL-1112) to measure snout-vent length (mm) and a digital weighing balance (BL 60001-5) to weigh individuals (grams). We ensured that the attachment assembly would not exceed 10% of the frog’s total body mass (Richards et al. 1994).

***Radio tracking.***—We installed radio transmitters on three Murree Hills Frogs (1 ♂, 2 ♀) and five Hazara Frogs (2 ♂, 3 ♀) in September 2017 and 10 Murree Hills Frogs (5 ♂, 5 ♀) and eight Hazara Frogs (6 ♂, 2 ♀) in September 2018 (non-breeding season) for eight consecutive days during each session. Details on specimens (sex, snout-vent length and weight) and transmitters are given in Table 1. Since the two studied frog species are nocturnal, we located and observed the tracked frogs three times, every three hours from sunset to sunrise. We recorded the distance moved by each frog and calculated mean distances (m) moved for males and females of each species for the entire session.

After testing normality of our data (*P* > 0.05 for Shapiro-Wilk test) in SPSS 25, we used the Mann-Whitney test to compare distances (median) moved by males and females of each species in a given year and distances moved by radio-tracked frogs (pooled data for males and females) of each species between 2017 and 2018 (α = 0.05).

## Results

We did not observe much movement (limited to < 3 m) along the stream or away from the stream into the forest by either species. The mean distance (m) moved by radio-tracked males and females of Hazara Frogs and Murree Hills Frogs in 2017 and 2018 is given (Table 1). We lost a few transmitters (*Allopaahazarensis*, ♂ = 4 , ♀ = 1; *Nanoranavicina*, ♂ = 2, ♀ = 3) during the study period and, consequently, distance data were not recorded in these cases (Table 1). We found a significant difference between the distance (median) travelled by Murree Hills Frogs (pooled data for the two sexes) (U = 27; *P* = 0.046) in 2017 and 2018 with more distance travelled in 2017 (n = 6; 0.60 m) as compared to 2018 (n = 19; 0.30 m). We did not find significant differences between any of the radio-tracked males and females of Hazara Torrent Frogs in 2017 (U = 0.00; *P* = 0.58), 2018 (U = 7.50; *P* = 0.32) and of Murree Hills Frogs in 2017 (U = 1.00; *P* = 0.10) and 2018 (U = 34.50; *P* = 0.43) and Hazara Torrent Frogs (pooled data for the two sexes) (U = 48; *P* = 0.31) between 2017 and 2018.

## Discussion

We provided data on the movement pattern of two frogs endemic to Himalayan region, Hazara Torrent Frog (*Allopaahazarensis*) and Murree Hills Frog (*Nanoranavicina*), for the first time. Our data showed that these frog species exhibit limited movement during the observed time period. A synthesis of review on movement and dispersal in amphibians by Rais and Ahmed (2021) suggests that movement and dispersal of short distance (< 1 km) are common, of medium distance (2-4 km) are uncommon and of long distances (> 5 km) are very rare. Funk et al. (2005) reported that only 4% of marked Columbia Spotted Frog (*Ranaluteiventris*) adults moved distances greater than 200 m. Berven and Grudzien (1990) reported only two Wood Frogs (*Ranasylvatica*) moved distances of 2,530 m. De Villiers and Measey (2017) reported that only 5% of marked African Clawed Frogs (*Xenopuslaevis*) made over-land movements with distances of ~ 150 m and only 91 individuals moved distances of 2.4 km. The limited extent of movement exhibited by the Murree Hills Frog and the Hazara Torrent Frog during the present study indicates that these endemic frogs depend on a specific stream to live and reproduce, which have critical implications for their conservation.

The Murree Hills Frogs and Hazara Torrent Frogs are facing anthropogenic threats, such as habitat degradation, urbanisation and natural threats, as well as climate change (Ohler and Dutta 2004, Khan et al. 2008). Accordingly, we propose a movement paradigm for these two frog species. In response to such anthropogenic threats and/or climate change, these frog species might face local extinction if they cannot move greater distances or move over-land through open forest to colonise nearby streams (which are, in most cases, > 300 m away from the studied area; Muhammad Rais, pers. obs). These species may have two options for the dispersal and establishment of a metapopulation:

1. move upstream, which would require use of energy reserves and may subject newly dispersed/immigrants to competition with individuals already inhabiting upstream areas,
2. move downstream into unsuitable habitat in lower elevations, with more urban settlements, pollution and deforestation.

**Figure 3.**
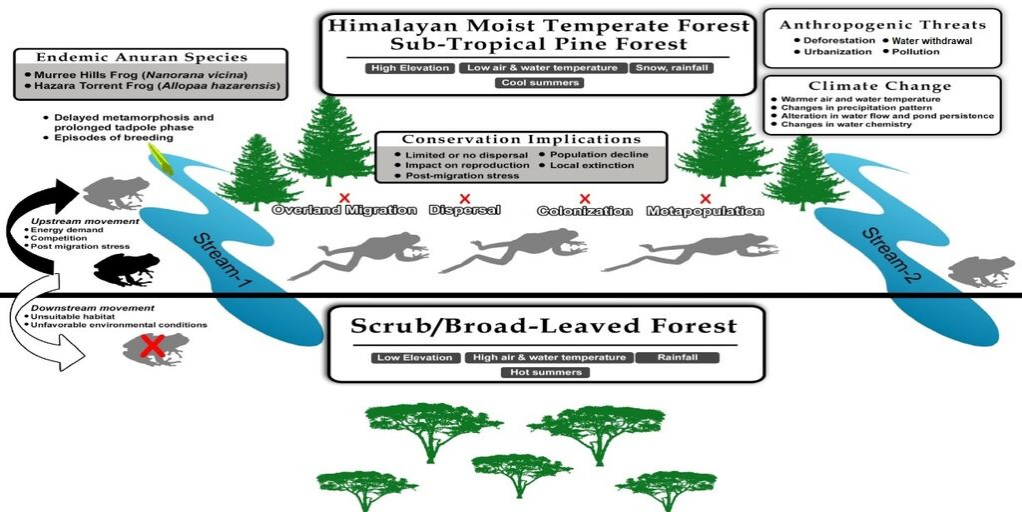
Movement paradigm for endemic frogs: Hazara Torrent Frog (*Allopaahazarensis*) and Murree Hills Frog (*Nanoranavicina*) in subtropical pine forest, based on our radio tracking data.

The species are not expected to take the risk of dispersal into subtropical scrub streams located further south due to unfavourable habitat and unsuitable environmental conditions. Increase in the air and water temperature or water withdrawal from the streams by the local community could seriously impact populations of these species. The species might be forced to perform over-land migration through the forest to occupy nearby streams, which are situated at a distance difficult to travel by amphibians or perform upstream migration that would require considerable energy reserves and may cause stress in the individuals (Fig. 3). Various mitigation approaches, such as construction of artificial wetlands, enhanced habitat connectivity and wetland restoration have been proposed to mitigate such effects elsewhere in the world (Brand and Snodgrass 2010, Lehtinen and Galatowitsch 2001, Rais and Ahmed 2021). These could be tested in the study area in future for the conservation of the two studied species.

We could have provided a more detailed data of movement in these frogs if we had not lost 38% of our fitted transmitters. The terrain is hilly and the stream had large boulders which were difficult to move. It was unclear whether the transmitters were lost while the frogs hid beneath the boulders, due to predation or to heavy rains which created flooding in the stream. For future research, we suggest using conventional mark-recapture study techniques or using PIT tags. It will reduce cost and more frogs could be included in the study. Likewise, we also suggest carrying out additional studies by incorporating multiple adjacent stream systems to better understand dispersal and colonisation by these frogs.

## Figures and Tables

**Figure 2a.**
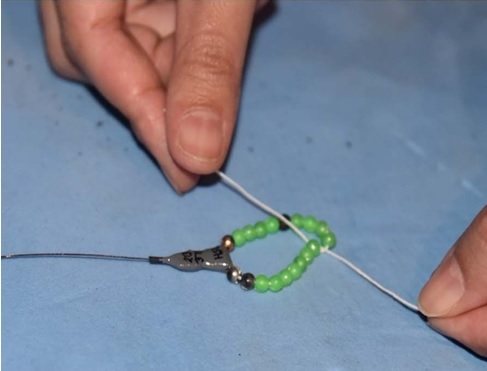


**Figure 2b.**
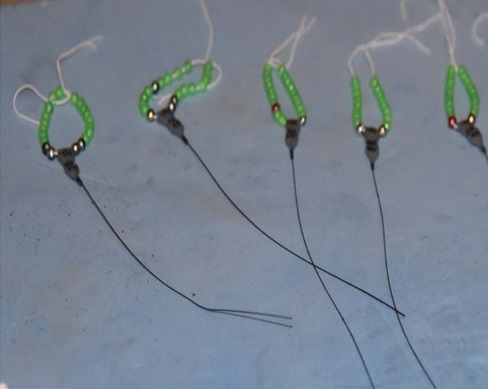


**Figure 2c.**
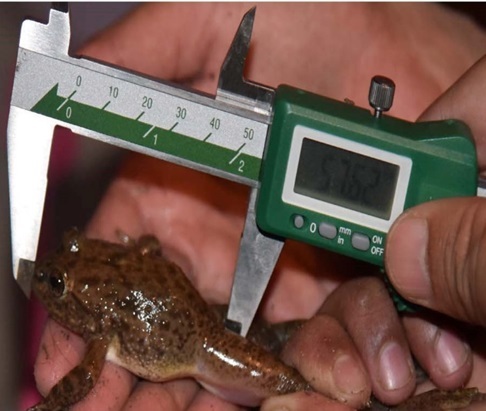


**Figure 2d.**
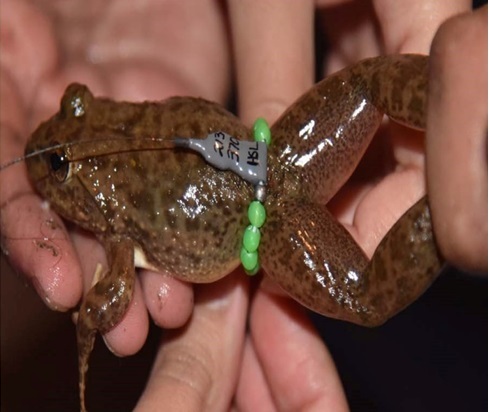


**Figure 2e.**
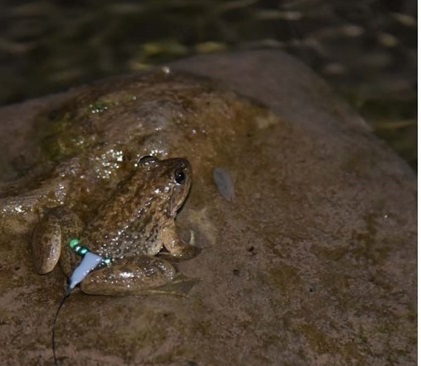


**Figure 2f.**
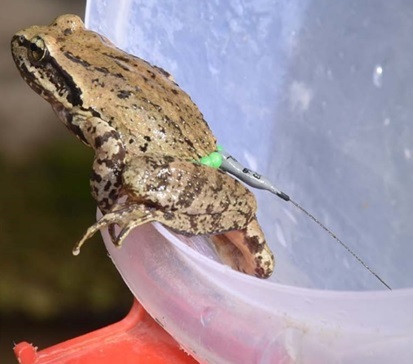


**Table 1.** Mean distance (meter) moved by Hazara Torrent Frog (*Allopaahazarensis*) and Murree Hills Frog (*Nanoranavicina*) along the studied stream in Village Parhanna, Tehsil Murree, District Rawalpindi, Punjab Province, Pakistan, during eight days in September 2017 and 2018. *missing data due to the loss of the transmitter

**2017**							
**Hazara Torrent Frog (*Allopaahazarensis*)**							
**Sex**	**ID**	**Transmitter Frequency (Hz)**		**Snout-vent Length (mm)**	**Weight (gm)**	**Distance (m)**	
♂	99	150. 712		58	152.82	1.5	
♂	97	150. 550		57	151.9	*	
					**Mean (♂)**	**1.5**	
♀	100	150. 755		59	159.04	1.51	
♀	98	150. 670		64	158.25	1.2	
♀	102	150. 867		75	176.4	2.41	
					**Mean (♀)**	**1.7± 0.31**	
**Murree Hills Frog (*Nanoranavicina*)**							
♂	101	150. 831		81	172.5	1.5	
					**Mean (♂)**	**1.5± 0.17**	
♀	103	150. 904		87	207.96	*	
♀	104	150. 948		98	256.3	2.72	
					**Mean (♀)**	**2.72± 0.30**	
**2018**							
**Hazara Torrent Frog (*Allopaahazarensis*)**							
♂	368	150. 593		56	158.5	0.6	
♂	99	150. 712		53	149.56	0.6	
♂	101	150. 832		50	153.9	1.5	
♂	373	150. 895		49	150.34	*	
♂	365	150. 396		36	140.2	*	
♂	367	150. 575		37	148	*	
					**Mean (♂)**	**0.9± 0.14**	
♀	370	150. 695		64	161.33	*	
♀	366	150.533		60	160.4	0.9	
					**Mean (♀)**	**0.9± 0**	
**Murree Hills Frog (*Nanoranavicina*)**							
♂	369		150.614	58	164.18		2.11
♂	374		150.975	79	189.08		1.5
♂	371		150.795	75	187.91		*
♂	100		150.755	89	222.35		*
♂	102		150.867	81	213.41		1.2
					**Mean (♂)**		**1.6± 0.27**
♀	104		150.949	73	173.05		*
♀	363		150.352	61	179.31		0.6
♀	372		150.813	72	187.85		0.6
♀	98		150.670	88	212.41		2.41
♀	362		150.313	74	173.05		*
					**Mean (♀)**		**1.2± 0.33**
